# Synaptophysin depletion and intraneuronal Aβ in organotypic hippocampal slice cultures from huAPP transgenic mice

**DOI:** 10.1186/s13024-016-0110-7

**Published:** 2016-06-10

**Authors:** Claire S. Harwell, Michael P. Coleman

**Affiliations:** The Babraham Institute, Babraham Research Campus, Cambridge, CB22 3AT UK; Present Address: John van Geest Centre for Brain Repair, Forvie Site, Robinson Way, Cambridge, CB2 0PY UK

**Keywords:** Alzheimer’s disease, Amyloid, Synapses, Organotypic brain slice, Intraneuronal Aβ, TgCRND8

## Abstract

**Background:**

To date, there are no effective disease-modifying treatments for Alzheimer’s disease (AD). In order to develop new therapeutics for stages where they are most likely to be effective, it is important to identify the first pathological alterations in the disease cascade. Changes in Aβ concentration have long been reported as one of the first steps, but understanding the source, and earliest consequences, of pathology requires a model system that represents all major CNS cell types, is amenable to repeated observation and sampling, and can be readily manipulated. In this regard, long term organotypic hippocampal slice cultures (OHSCs) from neonatal amyloid mice offer an excellent compromise between in vivo and primary culture studies, largely retaining the cellular composition and neuronal architecture of the in vivo hippocampus, but with the in vitro advantages of accessibility to live imaging, sampling and intervention.

**Results:**

Here, we report the development and characterisation of progressive pathological changes in an organotypic model from TgCRND8 mice. Aβ_1-40_ and Aβ_1-42_ rise progressively in transgenic slice culture medium and stabilise when regular feeding balances continued production. In contrast, intraneuronal Aβ continues to accumulate in close correlation with a specific decline in presynaptic proteins and puncta. Plaque pathology is not evident even when Aβ_1-42_ is increased by pharmacological manipulation (using calpain inhibitor 1), indicating that soluble Aβ species, or other APP processing products, are sufficient to cause the initial synaptic changes.

**Conclusions:**

Organotypic brain slices from TgCRND8 mice represent an important new system for understanding mechanisms of Aβ generation, release and progressive toxicity. The pathology observed in these cultures will allow for rapid assessment of disease modifying compounds in a system amenable to manipulation and observation.

**Electronic supplementary material:**

The online version of this article (doi:10.1186/s13024-016-0110-7) contains supplementary material, which is available to authorized users.

## Background

The progressive, permanent loss of neurons in Alzheimer’s disease (AD) means the potential for effective intervention declines as the disease advances [1]. Examining AD pathology at a late stage also makes identifying the primary drivers of the disease cascade challenging, as pivotal processes are masked by a melee of downstream consequences [2]. Taken together, it is clear that for effective therapeutics to be developed, we must observe, and target, the disease in its initiating phase. Synaptic dysfunction has long been noted as one of the earliest hallmarks of AD [2], occurring in advance of neuronal death [3, 4] and correlating best with soluble, rather than plaque-forming, Aβ [5]. Within as little as 2 years of clinical onset, cortical synapse density can be reduced by 35 % in patients [6], such loss of synapses being the best correlate of clinical outcome [7]. Presynaptic terminals seem especially vulnerable at this stage; synaptophysin [8], rab3a [9, 10], and synaptobrevin [10] are all significantly depleted in early AD brain tissue [11]. Despite other limitations, familial AD (FAD) mouse models recapitulate the synaptic alterations of the early stages of AD. As in human patients, there is a plaque-independent loss of synaptophysin-immunoreactive presynaptic boutons [12–14] as well as disruption of essential presynaptic components within early dystrophic axons [15]. Direct readouts of synaptic function also reveal deficits; LTP showing more rapid decay in FAD mice [16]. Such alterations occur in models lacking tau pathology, demonstrating alterations in Aβ processing, at least in the presence of wild-type murine tau [17], are sufficient to cause some synaptic deficits.

Investigation of the relationship between Aβ and early synaptic deficits would be facilitated by a model system amenable to repeated sampling, imaging and manipulation. While outer cortical layers of FAD mouse brain can be imaged using a cranial window [18, 19], key areas of pathology such as the hippocampus cannot. Instead, a major, terminal operation is needed to reach them, limiting observations to a single occasion [20]. Delivery of drugs is also limited by the blood brain barrier or by the acute and invasive nature of intracerebroventricular delivery. In vitro models, particularly of primary cortical neurons, bypass many of these issues, but fail to account for the complexity of the mammalian brain, its specialised neuronal circuits and variety of interacting cell types. Organotypic Hippocampal Slice Cultures (OHSCs) fill an important gap between these two systems. Whilst maintaining a complete hippocampal circuit and associated cell populations for several weeks, they allow for a level of experimental manipulation and accessibility that is unobtainable in vivo [21]. Screening for potential disease-altering compounds is made easier by the lack of blood-brain barrier or any systemic effects, and the ability to completely alter the extracellular environment in a matter of seconds. By adding (or removing) test compounds at different time points, their ability to prevent, halt or reverse pathology can be easily assessed. As such, OHSCs present an ideal platform to both observe and manipulate the amyloid cascade.

OHSCs from FAD mice have been surprisingly underused in the AD research field, especially to study progressive pathology caused by endogenously produced Aβ. Some studies have focused on applying synthetic Aβ (in various aggregation states) to acute cultures from non-transgenic animals. Such work has highlighted the importance of oligomeric Aβ in AD pathology, due to its enhanced ability to suppress of LTP [22], cause neuronal death [23] and alter dendritic spine density [24]. Other work has focussed on understanding plaque formation and dissolution, showing, for example, that microglia from adult FAD mice (when compared to juveniles or WT controls) are ineffective at clearing synthetic Aβ fibrils [25], and that neprilysin, insulysin and matrix metalloproteinases can aid clearance of plaques already present in slices prepared from adults [26]. Occasional studies using OHSCs from FAD mice have used a single timepoint to focus on dendritic spine or branching alterations following transduction with human tau [27, 28] or manipulation of tau pathology through pharmacological means [29]. There is also little known about presynaptic pathology in OHSCs, despite reports of extensive axonal swelling and synaptophysin depletion in FAD mice in vivo [15, 30] and in patients [31].

Here we describe an OHSC model from TgCRND8 mice [32] as a tool to explore the early and progressive effects of Aβ in central nervous system (CNS) tissue. We used this strain because amyloid pathology and axonal swelling begin within two months in vivo [15]. This is broadly within the timeframe for which OHSCs can be maintained [33], so we hypothesised that early stages of pathology caused by endogenous APP processing products should be detectable. We show that the transgenic slices are viable for in excess of 8 weeks, retain all major CNS cell types and rapidly release soluble Aβ into the culture medium, the production rate of which can be manipulated pharmacologically. While there is no plaque pathology, there is a striking accumulation of intraneuronal Aβ (at least some of it axonal), far more than in vivo, accompanied by a specific and progressive decline in presynaptic proteins. This is now an excellent experimental model for understanding the mechanism of progressive Aβ-induced synapse dysfunction and how it can be prevented.

## Methods

### Mice

TgCRND8 mice [32], overexpressing human APP with Swedish (K670N/M671L) and Indiana (V717F) FAD mutations, were maintained as heterozygotes on a 62.5:37.5 sv129: C57BL/6 background, generating transgenic and wild-type (WT) littermate controls. Thy1-mitoCFP [34] mice were also maintained as heterozygotes on a C57BL/6 background and females were crossed with TgCRND8 males to generate double transgenic mice. Animals were kept on a 12:12hs light: dark cycle at a constant temperature of 19 °C in a pathogen-free environment. All animal work was approved by the Babraham Institute Animal Welfare and Ethical Review Body and UK Home Office, and carried out in accordance with the Animals (Scientific Procedures) Act, 1986, under Project Licence 70/7620.

### Organotypic slice cultures

Organotypic cultures of the hippocampus and surrounding cortex were taken from humanely sacrificed P6-P9 mouse pups of either sex according to the method described by de Simoni et al. [33]. Briefly, brains were rapidly removed and kept in dissection buffer (EBSS+ 25 mM HEPES+ 1 X Penicillin/Streptomycin) on ice. From this point, until plating, all equipment and tissue was kept ice cold. Brains were bisected at the midline then the cut sides glued (Loctite), face down onto a vibratome stage and flooded with dissection media. 350 μm sagittal slices (6 per brain) were taken using a Leica VT1000S Vibratome; the hippocampus with surrounding cortex was dissected out using sterile syringe needles whilst the slice was lying on the vibratome blade. The dissected slices were then transferred (using a sterile 3 ml plastic pipette- modified to widen the opening) to Falcon tubes full of ice-cold dissection medium and stored until plating. To plate, slices were transferred (3 slices from the same brain per dish) onto sterile 0.4 μm pore membranes (Millipore PICM0RG50) in 35 mm culture dishes (Nunc). Inserts were kept in 1 ml of maintenance medium (50 % MEM with Glutamax-1 (Life Tech:42360-024), 25 % Heat-inactivated horse serum (Life Tech: 26050-070 ), 23 % EBSS (Life Tech: 24010-043), 0.65 % D-Glucose (Sigma:G8270) , 2 % Penicillin-Streptomycin (Life Tech: 15140-122)and 6 units/ml Nystatin (Sigma: N1638) )and cultures were maintained in incubators at 37 °C, 5 % CO_2_ for up to 12 weeks. Two 100 % medium exchanges occurred (5 hs after plating and 4 *div*) and a 50 % media exchange occurred each week thereafter.

### Aβ ELISA and drug treatments

To determine levels of human Aβ_1-40_ or Aβ_1-42_ in the slice culture medium, samples were analysed using commercially available ELISA kits (Life Tech: KHB3441/KHB3481). Briefly, culture medium was diluted to bring the expected concentration within the range of the standard curve before being incubated with Aβ detection antibody for 3 hs at room temperature. After washing, samples were incubated with HRP-conjugated anti-rabbit antibody for 30mins, washed, and then incubated with stabilised chromogen for 30mins. The reaction was stopped using an acid-based stop solution and absorbance read at 450 nm using a PheraStar FS plate reader. Samples were run with a standard curve (4-parameter fit) to obtain a concentration readout in pg/ml.

For quantifying Aβ within the slice tissue, material from a single culture membrane (3 slices) was homogenised in 10 μL 5 M Guanidine Hydrochloride supplemented with 1x Protease Inhibitor Cocktail (Roche) for 3-4 hs at room temperature (RT). The sample was then frozen at -20 °C until use. Prior to running in the ELISA, the homogenate was diluted 1:50 in ice cold reaction buffer (Dulbecco’s PBS + 0.03 % Tween + 5 % BSA supplemented with 1x Protease Inhibitor Cocktail) and centrifuged for 20mins at 4 °C at 16,000 *x g*. The supernatant was then diluted before undergoing ELISA readout as for the media samples. Readout is given as pg of Aβ per slice.

To determine how drug treatment influenced Aβ accumulation, slices were moved to fresh maintenance media for 24 hs. 50 μl of conditioned culture medium was then taken and frozen at -20 °C to act as baseline production readout. Calpain Inhibitor 1 (Sigma: A6185)) or DMSO control was then applied to the culture, with 50 μl of the treated medium dropped onto the slices to ensure complete drug infusion. The medium on top rapidly soaks through the slice, so the oxygen exchange is not hindered during this time. 50 μl aliquots of culture medium are then taken every 24 hs to monitor Aβ production, with readouts normalised to the original 24 h baseline. All drug experiments were run in triplicate, with 3 independent membranes from different mice used per experiment.

### Immunofluorescence staining

Membranes were transferred into 6 well plates and slices were fixed for 20mins in 4 % paraformaldehyde in 0.1 M PBS (applied both above and below the membrane insert). To reduce the volumes required for subsequent steps, the membranes were cut free of the plastic inserts and the sections of membrane containing the slices were transferred, using forceps, to individual wells in a 24 well plate. Slices were washed twice in TBS, blocked for 1 h in blocking solution (TBS with 0.5 % Triton X-100 and 3 % Goat Serum) then incubated in 200 μl primary antibody diluted in blocking solution overnight at 4 °C with shaking. Slices were washed 3 times in TBS before being incubated (2 hs, RT in the dark) with Alexa488, 568 or 647 conjugated secondary antibodies (Life Technologies-diluted 1:250 in blocking solution). After a final 3 TBS washes, some slices were counterstained with Thioflavin S, BTA-1, Nissl or Hoechst. Images were captured using a Nikon Confocal Microscope. Primary antibodies used: mouse Tuj1 (Covance 1:1000), rabbit Tuj1 (Sigma 1:500), chicken Tuj1 (Abcam:1:1000) rabbit NFL (Millipore 1:250), mouse MOAB2 (pan specific to Aβ- Millipore 1:1000), rabbit GFAP (Abcam 1:1000), mouse synaptophysin (Dako 1:1000), rabbit PSD95 (Abcam 1:500), rabbit tau (Dako 1:1000), rabbit Iba1 (Wako 1:500) rabbit calbindin and rabbit parvalbumin (Kind gifts from Dr P Emson 1:1000).

### Quantification of Aβ positive swellings

To quantify the degree of Aβ positive swellings in OHSCs of different ages, x20 images of MOAB2 staining in the CA1 region were captured (blinded to culture age and using identical microscope settings for each image) then processed using Fijix64 image analysis software [35]. The MOAB2 (red) channel was isolated and the image threshold manually adjusted to remove background (thresholding was performed blind to culture age, with the original image open in parallel, to ensure the thresholded image accurately represented visible staining). The plugin “despeckle” was applied to remove isolated pixel noise before the “Analyze particles” plugin was run. “Total particle count” results were compared between slices of different ages.

### Synaptic marker image capture, processing and puncta quantification

5-week old TgCRND8 and WT slices were imaged according to the synapse quantification protocol adapted from Ippolito and Eroglu [36–38]. Briefly, slices were stained for PSD95 (secondary labelled Alexa-568) and Synaptophysin (secondary labelled Alexa-488) according to the standard immunofluorescence protocol described above. Using a Zeiss 780 confocal x63 oil-immersion objective, image stacks from the CA1 (location of Aβ- positive axonal swellings in TgCRND8 cultures) and CA3 (largely swelling-free) fields were collected. For each slice, the chosen field was imaged using serial optical sections at 0.33 μm for a total of 15 sections (total depth of 5 μm). Maximal intensity projections (MIPs) were generated from 3 consecutive optical sections, resulting in 5 images each displaying 1 μm depth per field section in a slice. Quantification was performed using an imageJ 1.29 plugin [36, 39, 40] (available from c.eroglu@cellbio.duke.edu). Briefly, 33 μm x 33 μm regions of interest were randomly selected from each MIP and the “Puncta Analyzer” plugin run. Red (PSD95) and green (Synaptophysin) channels were manually thresholded to highlight visible puncta without the introduction of background noise. The plugin provides quantitative data for puncta number in each channel, as well as the number of colocalised puncta. 8 membranes per genotype (from different mice) were analysed, the MIP values from individual slices on the same membrane averaged to give an overall “membrane average”. Throughout image collection and analysis, the experimenter was blind to slice genotype.

### Western blotting

Slices were scraped off the membrane, treated with 2x Laemelli buffer + 10 % 2-mercaptethanol (250 μL per 3 slices) vortexed, boiled, then frozen at -20 °C until use. For use, most samples were further diluted 1:2 and loaded 8 μl per lane in a precast 4-20 % gradient gel (Bio-rad). To detect the weaker signal for PSD95, 15 μl of undiluted sample was loaded. After incubation in primary antibody overnight, blots were probed with 1:5000 mouse-700 (Life Technologies) and rabbit-800 (LI-COR) secondary antibodies then imaged using a LI-COR Odyssey detection system. Band intensity (IKK) was quantified using Odyssey software then normalised to Tuj1 signal. Primary antibodies used: mouse synaptophysin (Abcam: 1:1000), rabbit tuj1 (Sigma: 1:2500), rabbit PSD95 (Abcam: 1:500), mouse VAMP2 (Synaptic Systems: 1:10,000) and mouse RT97 (Kind gift from Dr Diane Hanger 1:500). Tuj1-normalised protein expression was then compared between WT and TgCRND8 cultures, TgCRND8 values expressed as a percentage of the WT average.

### Statistical analysis

Analysis was conducted using GraphPad Prism software. To assess calpain inhibitor dose response and Aβ swelling count data, one way ANOVAs with Dunnett or Tukey post hoc tests (respectively) were used. For synaptic protein western blot and calpain inhibitor treatment effects, two way ANOVAs with Sidak post hoc tests were conducted. For synaptic puncta counts, Student’s t-tests were used. Results are expressed as mean +/- standard error.

## Results

### Slices from TgCRND8 mice survive in excess of 7 weeks in vitro, demonstrate preserved architecture and possess the expected range of cell types

**Fig. 1:**
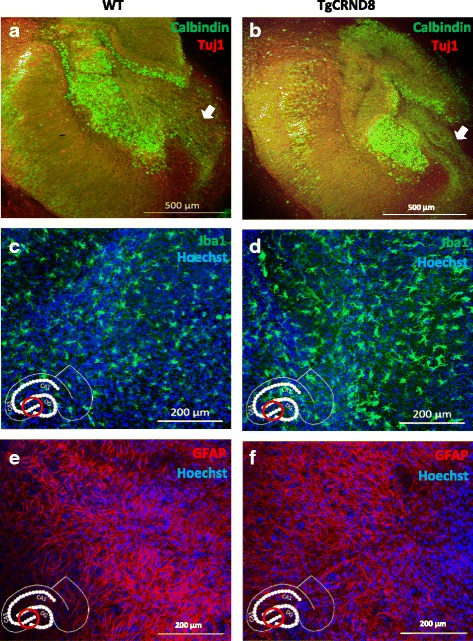
OHSCs maintain hippocampal architecture and express a full complement of different cell types. **a**, **b** 7-week old WT (**a**) and TgCRND8 (**b**) slices stained for Calbindin (*green Alexa-488*) and Tuj1 (*red Alexa-568*). Arrowheads show axons projecting from the dentate gyrus **c**, **d** 5-week old WT (**c**) and TgCRND8 (**d**) slices showing microglia (*Iba1- green Alexa 488*) and nuclei (Hoechst- *blue*) **e**, **f** 7-week old WT (**e**) and TgCRND8 (**f**) slices showing astrocytes (*GFAP-red Alexa568*) and nuclei (*Hoechst-blue*). Image locations, where appropriate, are denoted by the red ring on the hippocampus diagram inset in each panel

To demonstrate that the major architecture of the hippocampus is maintained throughout our experimental time-points, we fixed and stained slices at 7 weeks in vitro. We found the dentate gyrus and CA1 to be remarkably preserved in both WT (Fig. 1a) and TgCRND8 (Fig. 1b) cultures (as shown by Calbindin staining) with clear axonal tracts seen projecting from the dentate gyrus. Other major cell types are also well represented, with microglia (Fig. 1c, d) and astrocytes (Fig. 1e, f) clearly visible in both WT and transgenic slices.

### Aβ_1-42_ is continuously released into the culture medium

**Fig. 2:**
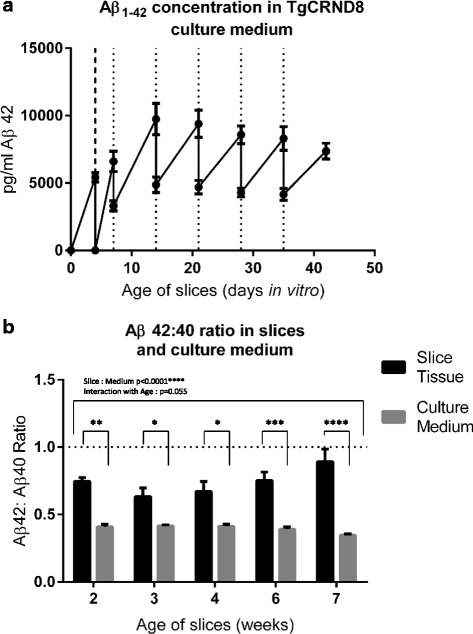
Measurement of Aβ in TgCRND8 slice culture medium and slice tissue. **a** Aβ_1-42_ concentration in the culture medium was measured over time in vitro*.* Samples were first taken 4 days after plating, when medium was completely replaced (*thick dashed line*). At 7 *div* a further sample was taken before a 50 % feed (*thin dashed line*). Samples were then taken at weekly intervals, shortly before each 50 % medium exchange. There is a rapid rise in Aβ_1-42_ in the first 2 weeks in culture (shown by the gradient of the line between feeds), which slows after this point. Between 14 and 42 *div* production rate and removal are fairly well balanced, such that concentration lies between 5, 000 and 10,000 pg/ml between feeds. This corresponds to 1.1-2.2nM Aβ_1-42_. (*n* = 5 membranes, each from a different mouse (biological replicates)) (**b**) Comparison of Aβ_1-40_ and Aβ_1-42_ in the culture medium and slices homogenised in 5 M guanidine. Whilst Aβ_1-40_ is the predominant species in both sample types, Aβ_1-42_: Aβ_1-40_ ratio is significantly higher in slice tissue than in the medium throughout the culture period (2 way ANOVA *p* < 0.0001) indicating a greater proportion of Aβ_1-42_ is retained within the slice. There is a trend to this ratio difference increasing with age (2 way ANOVA *p* = 0.055). Star values comparing slice tissue and culture medium represent multiple comparisons from the ANOVA analysis. (*n* = 4 membranes per timepoint/sample type. Membranes in each timepoint were from different mice)

By taking samples before each feeding time point, it can be seen that Aβ_1-42_ is promptly released into the culture medium. There is a rapid initial production of Aβ between plating and around 14 *div* (potentially due to the inflammatory reaction to the slice preparation) whereafter the generation rate gradually slows, possibly reflecting a decline in cell numbers not necessarily linked to the transgene (Fig. 2a). A balance between Aβ_1-42_ production and removal (through weekly 50 % medium exchange) is achieved between 14 and 42 *div*, resulting in a consistent weekly fluctuation of between 5,000 and 10,000 pg/ml between feeds (final average concentration). No human Aβ_1-42_ is detected in WT slice medium as expected (data not shown). This demonstrates that the transgenic slices actively produce soluble Aβ_1-42_ and are exposed to detectable levels throughout the culture period (equivalent to around 1.1-2.2nM). The final average concentration can be increased by either reducing the feeding frequency (we determined a 50 % medium exchange each week to be optimal for allowing both Aβ_1-42_ accumulation and adequate delivery of nutrients), by increasing the number of slices plated per membrane (data not shown) or by pharmacological intervention (below).

### Aβ_1-40_ predominates in the culture medium, but the 42:40 ratio is higher in slice tissue

We compared the ratio of Aβ_1-42_ and Aβ_1-40_ in both slice tissue and culture medium from TgCRND8 slices (Fig. 2b). The Aβ_1-42_ isoform is generally considered more pathogenic than Aβ_1-40_ and is the more abundant of these two species in adult TgCRND8 mouse brain [32]. As such, we were surprised to note that Aβ_1-40_ is the predominant species within the slice culture medium between 2 and 7 weeks in vitro. Interestingly, whilst Aβ_1-40_ remains predominant in the slice tissue, the Aβ_1-42_:Aβ_1-40_ ratio is significantly higher in slice tissue when compared to the culture medium (*p* < 0.0001), with a strong trend for this difference to increase with the age of the slice (p = 0.055) . The higher Aβ_1-42_:Aβ_1-40_ ratio in the tissue indicates that Aβ_1-42_ has a greater propensity to be retained within the slice. This could be due to the increased aggregation tendency of this isoform preventing escape into the medium. The Aβ_1-42_:Aβ_1-40_ ratio in the slices is, however, still less than is seen at equivalent timepoints in vivo (0.74 at 4 weeks, 0.93 at 6 weeks [32]) demonstrating a diversion from the in vivo pathogenic time course. This could be seen as the slice modelling only the earliest stages of the disease, or accumulation of Aβ_1-40_ occurring more in slice medium due to lack of a vascular system to clear it. We also found that the total Aβ detected in the slice tissue is only 0.1 % of the amount of Aβ detected in the medium (data not shown), implying that any Aβ produced is very rapidly released into the extracellular environment.

### Intra-axonal Aβ accumulation in TgCRND8 slices

**Fig. 3:**
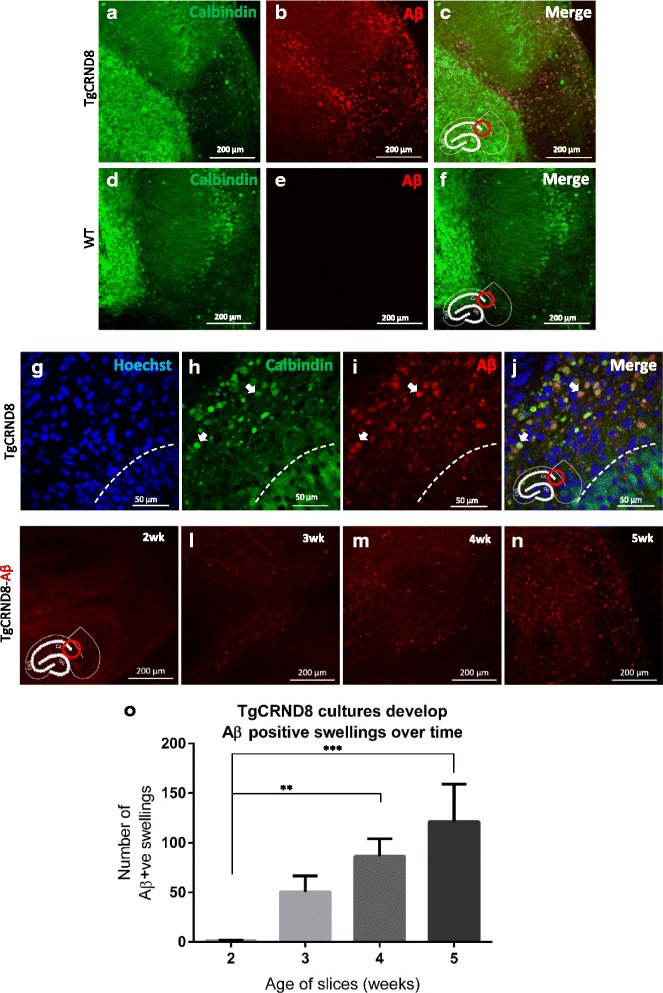
Intraneuronal Aβ accumulation in axons projecting from CA1. Where appropriate, image locations are denoted by the red ring on the hippocampus diagram inset in each panel. **a**-**c** Accumulation of Aβ in the CA1 region of a 5-week old TgCRND8 slice (**d**-**f**) no such Aβ staining is apparent in the same region in WT slices. **g**-**j** High magnification imaging of the Aβ containing region in a 6-week old old TgCRND8 slice. The cell bodies of CA1 (stained with calbindin) lie below the dashed line in these images. There is no strong colocalisation of Aβ with calbindin in this region, and no colocalisation with Hoechst, demonstrating lack of Aβ within the cell bodies of CA1. Above the dashed line lies the alveus, an axonal tract containing calbindin positive axons derived from CA1 (**h**). Many of these axons have large swellings, which do not colocalise with Hoechst (demonstrating these swellings are not cell bodies). There is however extensive colocalisation of these axonal swellings with Aβ (**i**) as indicated by the arrow heads. **k**-**n** 2,3, 4 and 5-week old TgCRND8 slices stained with MOAB reveal a progressive accumulation of Aβ positive swellings. **o** Quantification of Aβ positive swellings (total count) in 2-5-week old old slices. There is a significant increase in these structures over time (1 way ANOVA *p* < 0.05, *n* = 12 slices per timepoint (3 slices per mouse))

Previous studies indicate that intact axonal tracts are important for generation of Aβ [41, 42] and we reported a close association between axonal swellings and amyloid deposits as the disease progresses in mouse brain [15]. Thus, we then asked whether any of the Aβ present within the slice tissue is located within axons, as a possible precursor of the Aβ in the slice culture medium and of extracellular amyloid deposits in vivo. Immunofluorescence revealed that many TgCRND8 cultures develop extensive accumulations of Aβ as detected by MOAB2 (this antibody was specifically chosen as it is reported not to detect APP [43]) in vitro) (Fig. 3a-c). This is most abundantly seen in the regions adjacent to CA1 and is not seen in age-matched WT controls (Fig. 3d-f). Upon high magnification examination of the affected areas in TgCRND8 cultures, it can be seen that this Aβ staining colocalises with calbindin positive axonal swellings in the alveus (an axonal tract formed by projections from the cell bodies of CA1 [44–46]) (Fig. 3g-j). These swellings are relatively large (between 5 and 20 μm); much larger than the surrounding axon. There is no colocalisation of these swellings with Hoechst, further confirming (on top of their anatomical location) that these structures are not cell bodies. We examined the formation of these Aβ containing swellings in TgCRND8 cultures over time (Fig. 3k-n) and saw an increase in number between 2 and 5 weeks in vitro (Fig. 3o). It is important to note that WT slices also show calbindin positive swellings in the same region but do not show any sign of Aβ accumulation (Fig. 3d-f).

Staining with thioflavin S or BTA-1 (a sensitive thioflavin derivative) did not reveal convincing presence of extracellular Aβ deposition or plaque formation up to 12 weeks in vitro (data not shown). This is in contrast to the robust plaque development seen by 9 weeks in vivo [32] which is not accompanied by extensive intracellular Aβ staining. The conversion from extracellular to intracellular Aβ accumulation in TgCRND8 OHSCs represents an important difference between this model and in vivo.

### The production of Aβ_1-42_ is increased by calpain inhibition

**Fig. 4:**
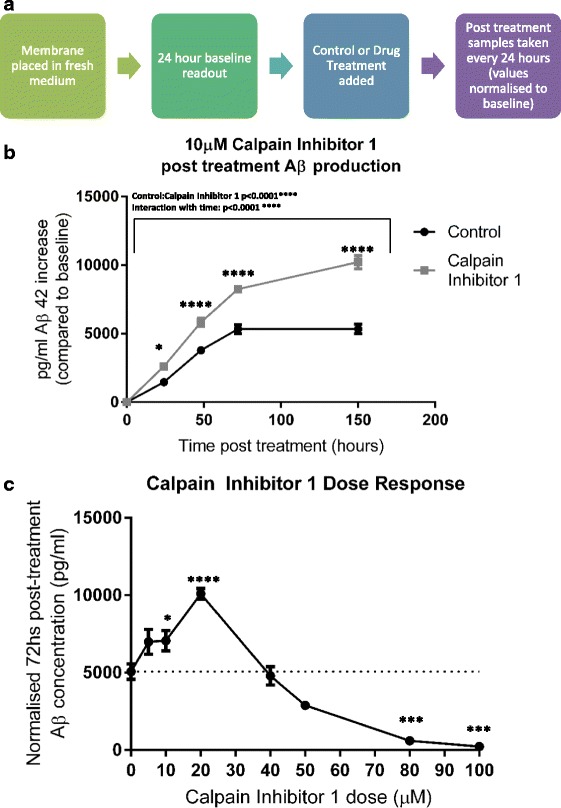
Treatment with Calpain Inhibitor 1 increases Aβ accumulation in TgCRND8 slice culture medium (**a**) Protocol for testing the effect of drug treatments. The slice membrane is placed in fresh maintenance medium and left for 24 hs. A 50 μL sample of medium is then taken to act as a “24 h baseline production” readout. Calpain Inhibitor 1 (or DMSO control) is then added to the culture, with treated medium allowed to soak through the slice from above. Timepoints are then taken at 24, 48, 72 and 150 hs post treatment and normalised to the 24 h baseline readout. (**b**) 10 μM Calpain Inhibitor 1 increases Aβ accumulation in slice culture medium to almost double that of control. (2 way ANOVA *p* < 0.0001(*Large bar above*) (stars on graph represent significance between treated and untreated at each timepoint via ANOVA multiple comparisons). Results are pooled from 3 independent experiments. *n* = 9 membranes per treatment (2 membranes arise from each mouse and are split between treated and control, so all samples within a condition are from different animals) (**c**) By changing dosage of Calpain Inhibitor 1 and sampling at 24 h baseline, then 72 h post treatment, a dose-response curve is revealed; 20 μM giving peak production, whilst concentrations above 40 μM reduce Aβ production to below that of control levels (1 Way ANOVA *p* < 0.05 (stars represent significant deviation from 0 μM treatment) n numbers: 0 μM = 11, 5 μM = 5, 10 μM = 12, 20 μM = 12, 40 μM = 5, 50 μM = 2, 80 μM = 3, 100 μM = 3. (Note n numbers are lower above 40 μM due to increased off-target toxicity in the culture system. All membranes used in each dose condition originated from different animals))

As there was no spontaneous plaque development in these OHSCs, we looked for ways to induce formation. We first sought to enhance the production of Aβ_1-42_ using one of the key advantages of slice cultures over in vivo models: the relative ease with which drugs can be delivered. A number of compounds were tested of which leupeptin, 27-hydroxycholesterol and LPS showed no consistent effect (data not shown). However, Calpain Inhibitor 1, which has previously been shown to increase Aβ production in APP overexpressing 293 cells [47], was more effective. We devised a protocol (Fig. 4a) that allows differences in Aβ production rate to be detected, by ensuring individual slice data is normalised to a pre-treatment baseline readout. Addition of Calpain Inhibitor 1 (10 μM) to 4-week old TgCRND8 slices approximately doubled the accumulation of Aβ_1-42_ in the culture medium relative to a DMSO control (*p* < 0.0001), with differences being apparent from as early as 24 hs after treatment (Fig. 4b). A dose-response curve in 2-week old slices indicated that 20 μM was most effective over 72 hs, whereas higher doses significantly reduced detectable Aβ below that of untreated cultures (Fig. 4c). However, no convincing plaque pathology was observed even after several weeks of 20 μM Calpain Inhibitor 1 treatment (data not shown). Attempts to seed pathology using exogenously applied synthetic Aβ also failed to elicit plaque formation (data not shown).

### TgCRND8 slices undergo age-dependent depletion of presynaptic proteins

**Fig. 5:**
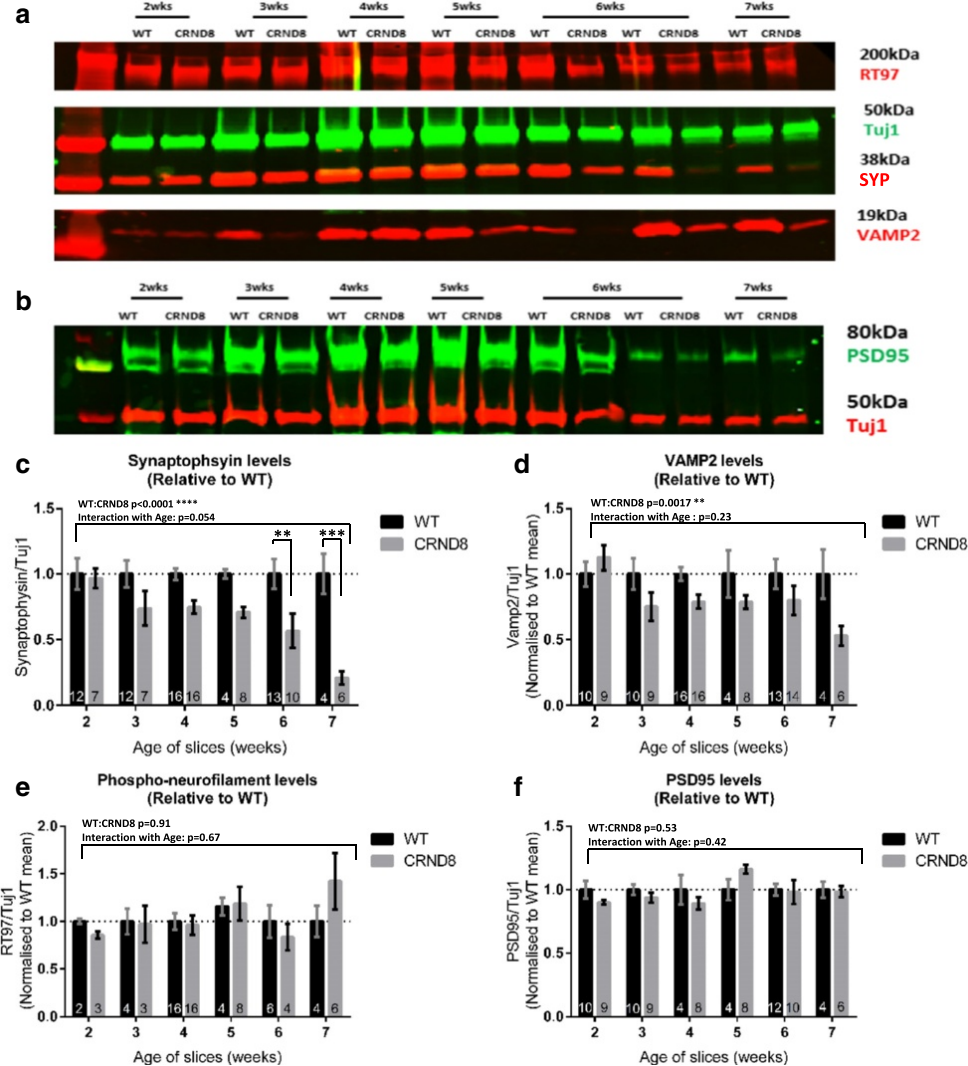
Quantification of synaptic protein levels using Western Blot. **a**, **b** Representative Western Blot of 2-7-week old WT and TgCRND8 slices probed for RT97 (phosphorylated neurofilament), Tuj1, Synaptophysin (SYP) and VAMP2 (**a**) or PSD95 and Tuj1 (**b**). **c** Synaptophysin levels (normalised to Tuj1) are decreased in TgCRND8 relative to WT (two way ANOVA *p* < 0.0001). There is a trend to this difference increasing with culture age (two way ANOVA *p* = 0.054), with 6 and 7-week old TgCRND8 cultures showing the greatest deficiency in synaptophysin (two way ANOVA multiple comparisons *p* < 0.05). **d** VAMP2 is also decreased in TgCRND8 cultures (two way ANOVA *p* = 0.0017) although the relationship with age is not clear (*p* = 0.23). There is no alteration in the levels of phosphorylated neurofilament (*p* = 0.91) (**e**) or PSD95 (*p* = 0.53) (**f**) demonstrating the losses seen are likely specific to presynaptic compartments. The large bar at the top of each graph displays tabular results from the two way ANOVA whilst individual bars on the graph show significant multiple comparisons. N numbers are written in the base of each bar, and represent the number of individual membranes (consisting of 3 slices extracted together and run in a single gel lane) from different mice

It is well established that synaptic deficits are one of the earliest events in Alzheimer’s Disease [2], and oligomeric Aβ is known to disrupt synapses [24, 48, 49]. Western blotting of slice homogenates from slices at 2-7 weeks in vitro (Fig. 5a, b) revealed a highly significant depletion of the presynaptic marker synaptophysin (normalised to the neuronal marker Tuj1) in TgCRND8 cultures when compared to WT (*p* < 0.0001) (Fig. 5c) that appears to worsen with the age of the slice (*p* = 0.054). VAMP2 was also reduced in TgCRND8 cultures when compared to WT (*p* = 0.0017) but the relationship with culture ages was less clear (*p* = 0.23) (Fig. 5d). As no such depletion was observed in the axon specific marker RT97 (*p* = 0.91) (Fig. 5e) or the post-synaptic marker PSD-95 (*p* = 0.53) (Fig. 5f), this suggests the deficiency is specific for presynaptic boutons rather than axons as a whole or both synaptic compartments.

### TgCRND8 slices show region specific reduction in pre-synaptic puncta and in PSD95/SYP colocalisation

**Fig. 6:**
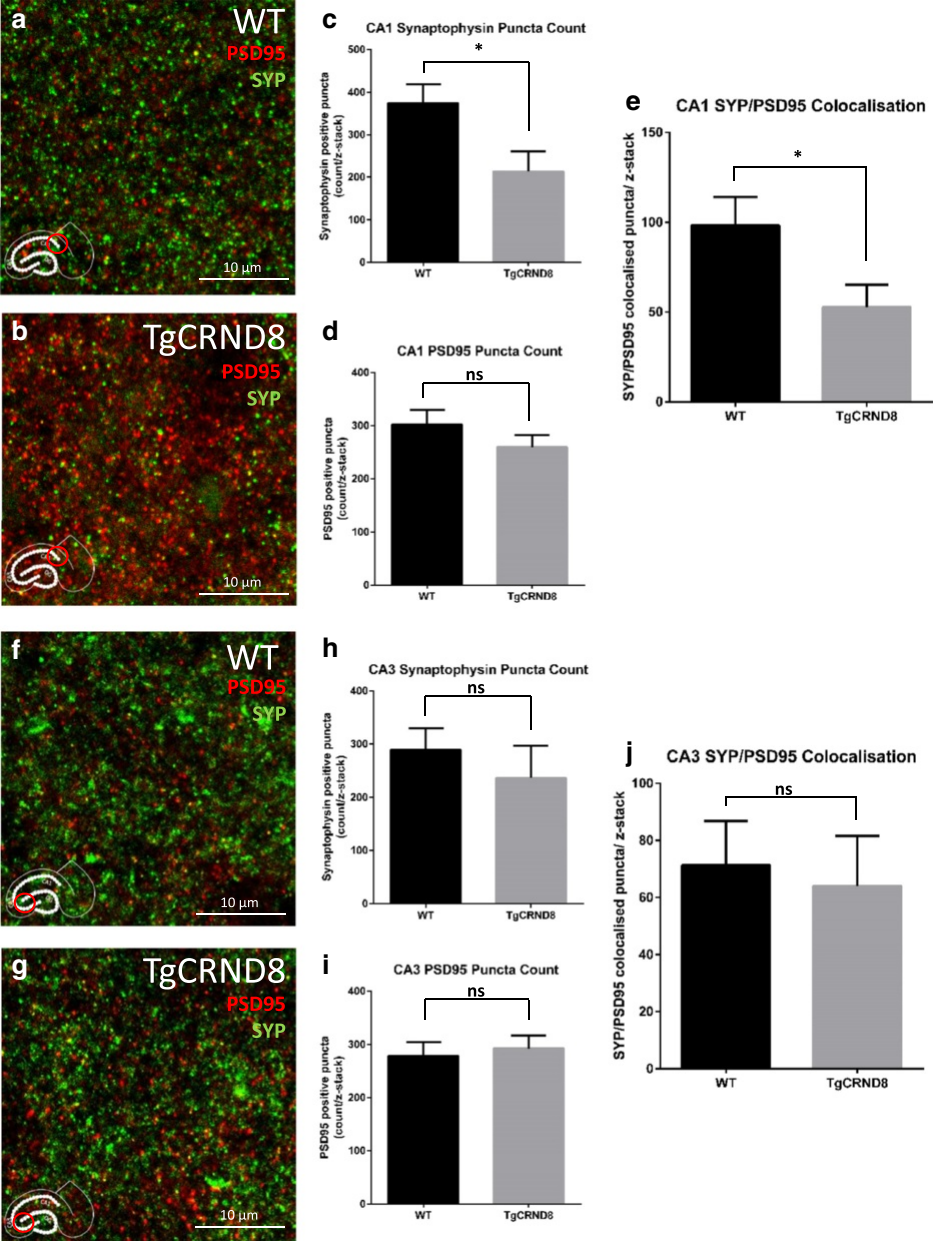
Region specific pre-synaptic puncta loss in TgCRND8 slices. **a**, **b** Representative maximal intensity projections of z-stacks taken from the CA1 field (same location as Aβ positive axonal swellings) in 5-week old WT (**a**) and TgCRND8 (**b**) slices. The presynaptic marker synaptophysin (SYP) is stained green, whilst the postsynaptic marker PSD95 is stained red. **c**, **d**, **e** Quantification of the z-stacks was performed using an image J plugin (available on request from: c.eroglu@cellbio.duke.edu). There was a reduction in synaptophysin positive puncta in TgCRND8 slices (*p* = 0.025) (**c**) whilst the number of PSD95 positive puncta was unchanged between genotypes (*p* = 0.25) (**d**). The number of colocalised puncta (Synaptophysin/ PSD95 positive) was also reduced (*p* = 0.038) (**e**). **f**, **g** Z-stacks taken from the CA3 field (largely unaffected by Aβ-positive swellings) in 5-week old WT (**f**) and TgCRND8 (**g**) slices revealed no such differences in synaptophysin positive puncta (*p* = 0.48) (**h**), PSD95 (*p* = 0.69) (**i**) or colocalised structures (*p* = 0.75) (**j**). Analysis for each region consisted of *n* = 8 individual membranes, 3 slices per membrane (slices on each membrane are from different mice). For each membrane value, data was averaged from 3 slices, with 5 z-stacks per slice) with *P* values calculated using a students t-test

As the appearance of Aβ containing axonal swellings correlates temporally with the loss of presynaptic proteins seen by western blot, we asked whether synaptic structures were specifically altered in the swelling-affected regions. By imaging presynaptic (synaptophysin) and postsynaptic (PSD95) puncta in both the CA1 (the region where Aβ positive swellings are found) and CA3 (a region lacking such structures) fields, we compared alterations in synaptic contacts (areas of co-localisation between pre- and post-synaptic marker labelled puncta [36, 50]). In the CA1 region (Fig. 6a, b) we found a significant reduction, compared to age-matched WT controls, in the number of pre-synaptic puncta in 5-week old TgCRND8 slices (*p* = 0.025) (Fig. 6c) without a corresponding decrease in PSD95 positive, post-synaptic puncta(*p* = 0.25) (Fig. 6d). This translated to a reduction in the number of PSD95/SYP colocalised puncta, consistent with a possible loss of synapses (*p* = 0.038) (Fig. 6e). In the CA3 region (Fig. 6f, g), no such alterations were apparent in either presynaptic puncta count (*p* = 0.48) (Fig. 6h), postsynaptic count (*p* = 0.69) (Fig. 6i) or PSD95/SYP colocalisation (*p* = 0.75) (Fig. 6j). Thus, the reduction in synaptophysin we observed in western blots at least partially reflects losses in presynaptic puncta within CA1.

## Discussion

Here we describe how long term OHSCs from TgCRND8 mice can be used as a model of Aβ pathology, revealing novel aspects of the disease mechanism that are not easily studied in vivo. We can now explore, in a system highly amenable to manipulation and analysis: release of Aβ, loss of presynaptic proteins, loss of SYP/PSD95 colocalisation and the accumulation of intraneuronal Aβ. We have also seen that addition of calpain inhibitor 1 enhances Aβ production in this model, potentially opening doors to examine how pathological outcomes change with rising Aβ. Whilst the nature and time course of pathology shows some interesting differences from that seen in vivo, long term OHSCs from TgCRND8 mice represent an exciting new tool for research into the early consequences of progressive accumulation of Aβ and other consequences of APP processing.

## Loss of presynaptic proteins and puncta correlates spatially and temporally with the appearance of intraneuronal Aβ

Loss of presynaptic proteins is thought to be one of the earliest (and most clinically relevant) changes in human AD [2, 51] with some reports indicating it precedes extensive postsynaptic changes [52]. In TgCRND8 OHSCs, the presynaptic proteins synaptophysin and VAMP2 are depleted when compared to WT controls. This corresponds to a region-specific loss of presynaptic puncta, consistent with loss of synapses. Our finding that PSD-95 protein and puncta are not depleted supports the notion that presynaptic changes can precede other alterations [52–54]. The preservation of axon-specific phosphorylated neurofilament also supports localised changes in the immediate presynaptic compartment, and not a wider loss of axons at this stage.

A key future direction will be to determine the cause of the presynaptic changes in the TgCRND8 slices. As plaques do not develop during the experimental timecourse (see below), they are not needed to drive synaptic alterations. Indeed, the lack of tau pathology in TgCRND8 mice also rules out neurofibrillary tangles as a necessary cause of the presynaptic protein depletion we see. Whilst the observed synapse effects are clearly dependent on the huAPP transgene, it is important to remember that OHSCs undergo a period of synaptic reorganisation in culture following the significant tissue injury at the time of slice generation that does not occur in vivo. In WT rat OHSCs, it has been shown that within the first 2 weeks in vitro, the maximal evoked EPSP increases, plateauing between 10 and 15 days in vitro, likely representing an increase in synaptic connections [55]. APP overexpression has been shown to have a negative effect on synapse development in vitro [56, 57] so it is possible that the TgCRND8 slices have a reduced capacity for this repair that at least partly underlies the lower presynaptic protein levels.

A clear difference between the WT and TgCRND8 OHSCs that associates both temporally and spatially with the synaptic changes is the gradual appearance of Aβ-containing swellings, most commonly located in the alveus adjacent to CA1. There is an increase in the presence of such structures between 2 and 5 weeks in vitro, which closely parallels the development of presynaptic protein deficiency. The loss of synaptic structures, as measured by PSD95/SYP puncta colocalisation, also spatially correlates with the regions affected by Aβ positive swellings. Previous studies have shown that intraneuronal Aβ accumulation in synaptic compartments is directly associated with abnormal cellular morphology [58] whilst another study found that reducing the intraneuronal pool of Aβ through synaptic activation was protective against loss of synaptic proteins [59]. The role of intraneuronal Aβ in pathogenesis has been a controversial topic [43, 60], so the experimental system we report, in which Aβ generation and its release through synaptic activity can be readily manipulated, will be useful in resolving its importance in the disease process.

## Intraneuronal Aβ in axonal swellings

Whilst many FAD mouse models have been reported to develop pre-plaque intraneuronal Aβ pathology, [60–63] it is particularly interesting that the appearance of such striking Aβ positive swellings in TgCRND8 OHSCs differs from the corresponding in vivo phenotype in these animals. Whilst adult TgCRND8 mice usually show APP immunoreactivity within dystrophic axons [15] and there is some evidence for lysosomal Aβ accumulation in older mice [64], extensive intra-axonal accumulations such as the ones found in the OHSCs are not seen at any age studied in this strain [15] (Additional file 1: Figure S1). Interestingly, a similar phenomenon has been reported in whole brain slices from adult APP_SDI mice; a failure to develop plaques, but appearance of atypical intraneuronal Aβ staining [26]. This suggests that a hippocampus in slice culture is more prone to retain Aβ within cells than in vivo so it will be interesting to investigate the mechanism that underlies this difference. The appearance of swellings in WT cultures (albeit without Aβ accumulation) could indicate this is a result of axonal damage during the slice procedure, or another consequence of the slice culture system. Similarly, intraneuronal Aβ accumulation is commonly observed in axonal injury after brain trauma. Studies in humans [65], pigs [66] and rats [67] have all reported that injured, swollen or broken axons can act as sites of Aβ accumulation- particularly in axonal end bulbs. The nature of the OHSC is such that axotomy of certain populations of neurons is unavoidable. Whilst there is evidence for re-organisation and recovery in this model [68], it may be that in the TgCRND8 cultures (which will already have elevated levels of both APP and Aβ) this injury further seeds accumulation of Aβ. However, as it takes over 3 weeks for the intraneuronal Aβ to accumulate, any link to the initial injury appears likely to involve additional steps.

A further possibility is, due to lack of sequestration in plaques, the OHSCs may be bathed in relatively high concentrations of *soluble* Aβ when compared to adult brain. There is evidence to suggest that neurons can actively uptake Aβ, with the axon being highlighted as a potential point of entry [69]. Indeed, application of synthetic Aβ to OHSCs from WT rats resulted in intraneuronal accumulation of this peptide in CA1 [70], the same region that is heavily affected in our TgCRND8 OHSCs. Perhaps this region is rendered more vulnerable during the slice procedure resulting in enhanced uptake of exogenous Aβ than would be seen in vivo.

Whatever the cause of the intraneuronal Aβ accumulations in OHSCs, the fact that it differs from the in vivo phenotype will help us to understand the factors that govern the balance between intracellular and extracellular amyloid pathology. By studying the balance between Aβ in the tissue and the medium it should also be possible to investigate factors that influence its rate of extrusion. As both aspects of pathology are present in sporadic human AD [58, 71, 72] understanding the mechanisms of each could assist in developing effective therapeutic interventions.

## TgCRND8 OHSCs do not develop plaques

An unexpected finding in TgCRND8 OHSCs is that plaques fail to develop even after 12 weeks in vitro, over 4 weeks after such pathology would develop in vivo [15, 32]. A potential explanation for this is that the large volumes of culture medium relative to the small quantity of slice tissue washes Aβ from the slices more effectively than vascular perfusion, preventing the seeding of plaques. Alternatively, it could be that the microglia in the OHSCs are more effective at preventing plaque formation, perhaps as a result of activation from the initial slice preparation. A recent paper demonstrated that microglia from juvenile 5xFAD mice or WT controls are highly effective at clearing synthetic Aβ fibrils applied to WT OHSCs, whilst adult 5xFAD microglia cannot prevent the formation of aggregates [25]. It may be that in the long term OHSCs, a more juvenile microglial phenotype is maintained, thus preventing plaque deposition. Understanding exactly how the slice system differs to in vivo will, once again, assist in unpicking the mechanisms behind AD pathology.

## A model for studying pre-plaque Aβ dynamics

Whilst plaque development is not observed, TgCRND8 OHSCs’ rapid and sustained production of soluble Aβ peptides is easily detected. This system is ideal for examining the effects of early rises in Aβ on the hippocampus in a pre-plaque forming stage. We have also demonstrated that the production rate of Aβ in OHSCs can be bidirectionally manipulated through pharmacological means. Addition of 20 μM Calpain Inhibitor 1 doubles Aβ in the medium relative to control, whilst addition of 50 μM or more supresses production (consistent with previous findings in APP transfected 293 cells [47]). Whilst therapies would seek to reduce the rate of accumulation of Aβ there are clear experimental advantages in being able to increase its generation to explore mechanisms in a model system. As well as potentially permitting earlier observation of defects in culture, it allows determination of whether the severity directly correlates with soluble Aβ concentration in the environment or whether changes proceed to completion after Aβ passes a threshold level. This distinction is vital for effective therapeutic targeting.

## Additional uses of the model

The OHSC model we describe has many potential uses in studying the mechanism of amyloid pathology and developing therapeutics, but also a number of limitations. Slice preparation involves massive tissue damage, resulting in axotomy, cell death and activation of inflammatory cells. Plaque pathology does not develop, and there is increased intra-axonal Aβ beyond that seen in vivo. There is also a gradual over proliferation of non-neuronal cells, no vascular system and no electrical input from other brain regions. It should also be remembered that the slices are generated from juvenile mice, where many cells have yet to reach their mature phenotypes.

However, the progressive changes we report in long term TgCRND8 OHSCs have significant potential to be of use to the AD research field. For example, the rapid production of soluble Aβ species from these slices could be utilised as a source of pathogenic amyloid peptides without presupposing which of them is/are the most toxic. These could then be introduced exogenously into other experimental systems such as primary neuronal cultures, or OHSCs of a different genotype, avoiding the use of supraphysiological concentrations of synthetic peptides. This system should also enable studies examining the spread of pathological proteins, and screening, at least at a secondary stage, for compounds that will block Aβ generation or release, or block synaptophysin depletion.

The ability to repeatedly image, or live image, OHSCs is also important. Unlike the in vivo hippocampus, which is difficult to image even using multiphoton microscopy, we have been able to observe live cells such as microglia (stained using isolectin B4 conjugated to Alexa fluora568 (Life Technologies)) (Additional file 2: Figure S2a) and the axonal transport of mitochondria in OHSCs expressing mito-CFP (Additional file 3). We find that axons from the dentate gyrus are easily located with this genetic label (Additional file 2: Figure S2b-c) and repeat imaging of the same slices is possible. Mitochondrial or other axonal transport dynamics have been implicated many times in the pathogenesis of AD. Further work will be needed to develop this transport imaging for quantitative assessment, but probing for differences in the TgCRND8 slices has the potential to enhance understanding in a way that would not be possible in vivo.

## Conclusions

In summary, we report the first characterisation of progressive deficiencies in OHSCs from a huAPP mouse model. This reveals both similarities and differences from observations made in the same mouse strain in vivo, thus validating this system as a model for some aspects of pathogenesis. The model will be particularly useful for understanding disease mechanism, both because it can be readily manipulated, repeatedly sampled and imaged, and because the observed differences from in vivo pathology provides a basis for understanding why these occur (for example, the shift from plaques to intraneuronal Aβ). This experimental system also has important potential as a drug-screening platform. Here, candidate drugs can be readily delivered and monitored, and their effects on all relevant cell types and neuronal circuits observed, thus filling a vital gap between primary culture and in vivo studies. We expect this OHSC model will find many applications in AD research.

## Abbreviations

AD, Alzheimer’s Disease; Aβ, Amyloid-beta; CNS, Central Nervous System; MIP, Maximum Intensity Projection; OHSC, Organotypic hippocampal slice culture; PSD95, Post synaptic density 95; SYP, Synaptophysin; VAMP2, Vesicle-associated membrane protein 2; WT, Wild-type

## Additional files

Additional file 1: Figure S1. MOAB2 immunostaining in a 14 month old TgCRND8 hippocampus. Aβ in extracellular plaques is stained using the MOAB2 antibody **(a)** and neurons in CA1 stain positively for calbindin **(b)** but there is no evidence for intra-axonal Aβ in the merged image **(c)**. (PPTX 3615 kb) Additional file 2: Figure S2. Demonstrations of live imaging capability in the TgCRND8 OHSC system. Live microglia labelled with IB4 conjugated to Alexa 568 **(a)** Mito-CFP labelling in TgCRND8x MitoP OHSC **(b)**. The dentate gyrus and axons projecting from it are clearly labelled. Multiphoton image of MitoCFP labelled neuron in OHSC **(c)**. (PPTX 749 kb) Additional file 3: Live axonal transport of mitochondria in a TgCRND8x MitoP OHSC. (1 frame per second capture rate, 80 frames per second playback rate). (AVI 9113 kb)
