# Supplementary figures and images for: Synaptophysin depletion and intraneuronal Aβ in organotypic hippocampal slice cultures from huAPP transgenic mice
Source: Mol Neurodegener. 2016 Jun 10;11:44. doi: 10.1186/s13024-016-0110-7 (PMC4903008; doi:10.1186/s13024-016-0110-7)

## Slide 1
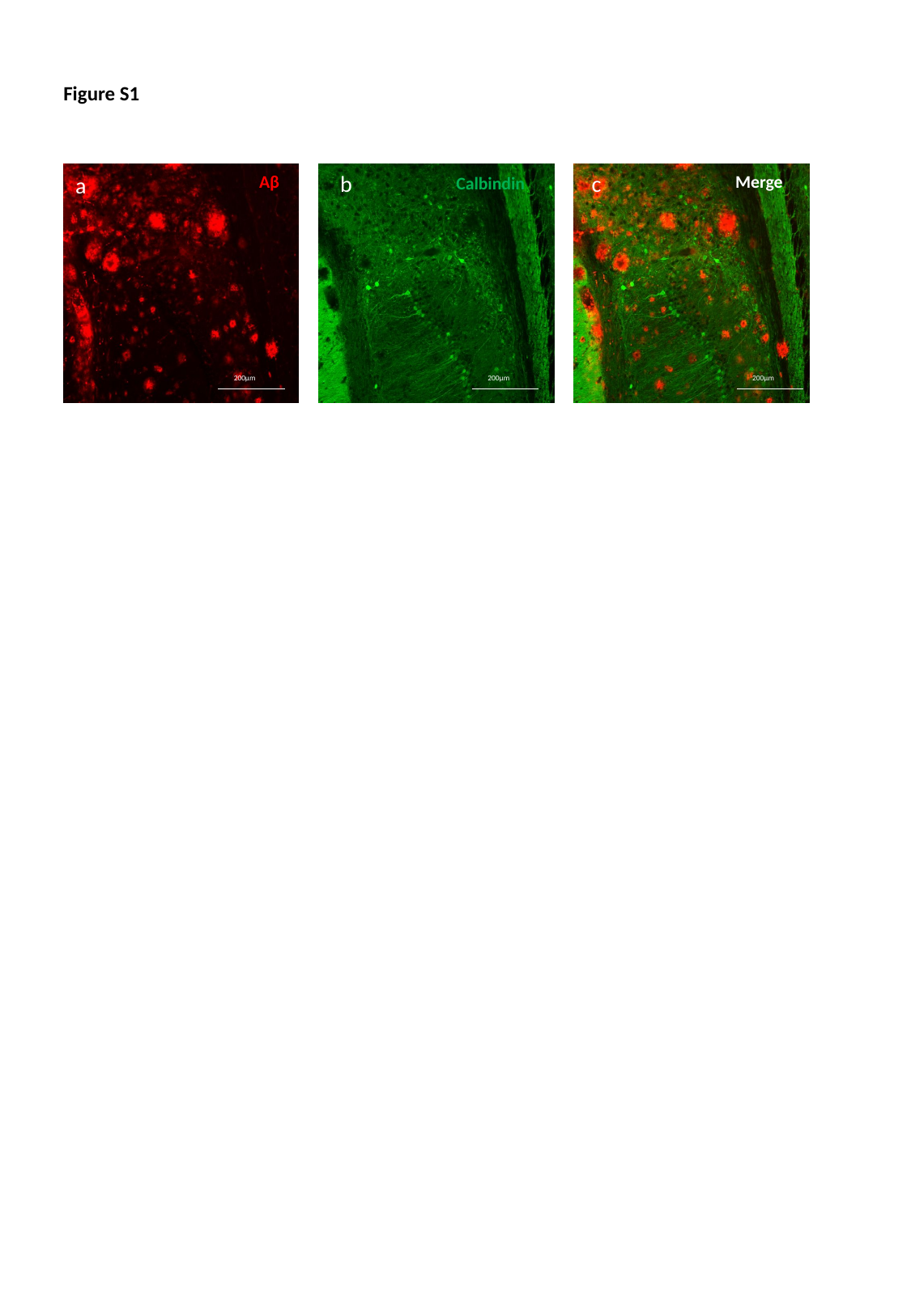

Figure S1
b
c
Merge
200µm
Aβ
200µm
Calbindin
200µm
a

Supplement: Additional file 1: Figure S1. — MOAB2 immunostaining in a 14 month old TgCRND8 hippocampus. Aβ in extracellular plaques is stained using the MOAB2 antibody (a) and neurons in CA1 stain positively for calbindin (b) but there is no evidence for intra-axonal Aβ in the merged image (c). (PPTX 3615 kb) [file 13024_2016_110_MOESM1_ESM.pptx]

## Slide 1
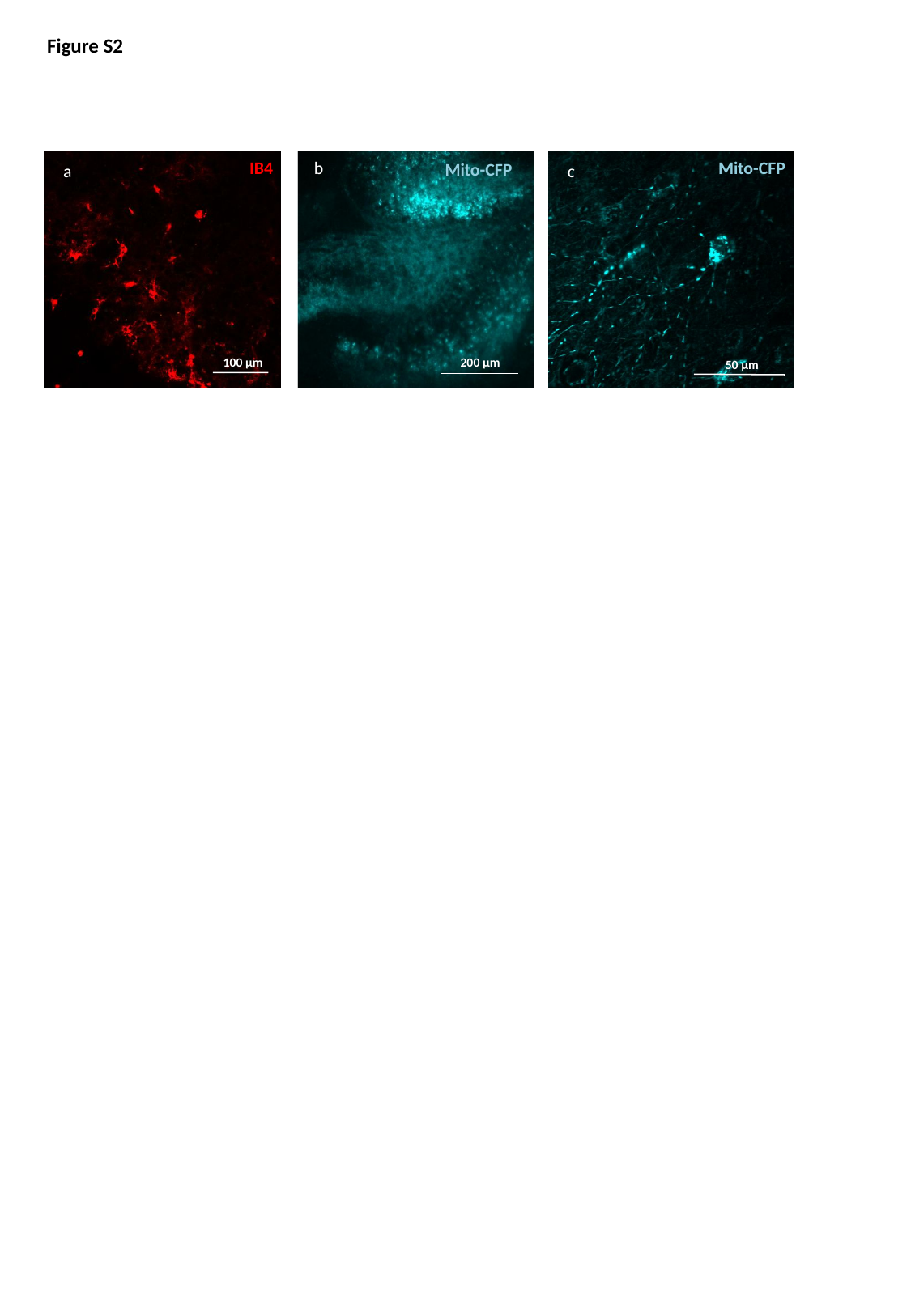

Figure S2
IB4
Mito-CFP
a
50 μm
c
Mito-CFP
100 μm
200 μm
b
b

Supplement: Additional file 2: Figure S2. — Demonstrations of live imaging capability in the TgCRND8 OHSC system. Live microglia labelled with IB4 conjugated to Alexa 568 (a) Mito-CFP labelling in TgCRND8x MitoP OHSC (b). The dentate gyrus and axons projecting from it are clearly labelled. Multiphoton image of MitoCFP labelled neuron in OHSC (c). (PPTX 749 kb) [file 13024_2016_110_MOESM2_ESM.pptx]
